# Distribution of Mast Cells in Uterine Leiomyoma: A Hospital-Based Observational Study

**DOI:** 10.7759/cureus.104841

**Published:** 2026-03-07

**Authors:** Sneha Soney, Hema Shantagiri

**Affiliations:** 1 Pathology, Jawaharlal Nehru Medical College, Karnataka Lingayat Education (KLE) Academy of Higher Education and Research (KAHER), Belagavi, IND

**Keywords:** hyaline degeneration, leiomyoma with degeneration, mast cells, myometrium, toluidine blue, uterine leiomyoma, uterine leiomyomata

## Abstract

Introduction

Mast cells are immune cells of the myeloid lineage present in connective tissues that regulate various physiological processes. The human uterus is relatively rich in mast cells. Uterine fibroids are the most common benign tumors, originating in the myometrium. However, there is no consensus regarding the prognostic role of mast cells in the uterus. Hence, further study is necessary for clarifying the relationship between the distribution of mast cells and various conditions of the myometrium.

Aims and objectives

The objective of this study was to quantify mast cell density in uterine leiomyomas compared with adjacent myometrium and to evaluate its association with tumor location, patient age, and degenerative changes.

Materials and methods

This was a retrospective and observational study conducted from January 2019 to December 2021 in the Department of Pathology, Jawaharlal Nehru Medical College, KAHER, Belagavi. Tissue blocks of hysterectomy patients operated for uterine leiomyoma, a total of 100 cases, formed the source of data. Two sections each were taken from the leiomyoma and its adjacent myometrium, processed routinely, and stained with hematoxylin and eosin and 1% toluidine blue. The mast cells were counted under 40× magnification for 10 consecutive fields in each slide.

Results

Leiomyomas showed a perimenopausal age preponderance, and 59% were intramural in origin. A statistically significant inverse association was observed between mast cell density and advancing age in leiomyomas. Compared to the adjacent myometrium, uterine leiomyomas demonstrated an approximately twofold increase in mast cell counts. Additionally, leiomyomas with degeneration were associated with lower mast cell counts.

Conclusions

Progressively decreasing mast cell counts were observed as we moved from the uterine leiomyoma to the adjacent normal myometrium. Leiomyomas with degeneration were characterized by a significantly lower mast cell density.

## Introduction

Mast cells (MCs) are immune cells of the myeloid lineage, present in connective tissues, which mature under the influence of the c-kit ligand and stem cell factor in the presence of other growth factors. Regulation of various physiological processes, including vascular homeostasis, innate and adaptive immune response, and angiogenesis, has been attributed to MCs. They have also been implicated in the pathophysiology of allergy, anaphylaxis, gastrointestinal disorders, and malignancies [1]. Notwithstanding mounting evidence of MC accumulation in tumors, their exact role in tumorigenesis and progression is poorly understood [2].

The human uterus is considered to be relatively rich in MCs compared to other tissues of the body. The greatest number was found in the inner (luminal) surface of the myometrium, while the endometrium contained significantly fewer MCs [3].

Uterine fibroids are among the most common benign tumors affecting women, originating from the uterine smooth muscle cells (myometrium). Although uterine leiomyomata are well recognized as hormone-dependent tumors, the contribution of immune and stromal components to their pathogenesis remains incompletely understood [4].

Several studies have reported increased MC density in uterine leiomyomas compared to normal myometrium. However, the available literature shows differences in methodology, quantification techniques, and sample size. Furthermore, limited data exist regarding the association of MC density with age, tumor site, and degenerative changes within leiomyomas.

Given these inconsistencies and the gaps in existing evidence, the present study was undertaken to evaluate the distribution of MCs in uterine leiomyomas, compare their density with that of the adjacent myometrium, and assess their association with various clinicopathological parameters, including age, site, and degeneration.

## Materials and methods

This was a retrospective and observational study conducted from January 2019 to December 2021 in the Department of Pathology, Jawaharlal Nehru Medical College, Karnataka Lingayat Education (KLE) Academy of Higher Education and Research (KAHER), Belagavi. The study was approved by the Institutional Ethics Committee of Jawaharlal Nehru Medical College, Belagavi (Approval No: MDC/DOME/120). As this was a retrospective study on archived specimens, informed consent was waived.

All consecutive hysterectomy specimens diagnosed with uterine leiomyoma during the study period (January 2019 to December 2021) were retrieved from the departmental archives. From the eligible cases, 100 cases were selected using a computer-generated random number sequence to minimize selection bias. These tissue blocks formed the source of data. Excluded from the study were patients on hormonal therapy, including oral contraceptive pills, and those with adenomyosis.

From each case, one representative formalin-fixed paraffin-embedded tissue block containing both leiomyoma and adjacent grossly normal myometrium was selected. The adjacent myometrium was defined as non-tumorous myometrial tissue located at least 5 mm from the tumor margin to avoid peritumoral reactive changes. Two sections, each 4 µm thick, were taken from the leiomyoma and its adjacent myometrium, processed routinely, and stained with hematoxylin and eosin and 1% toluidine blue.

MCs can be stained with hematoxylin and eosin, toluidine blue, Giemsa, MC tryptase, chymase, and CD117, although studies have revealed that the best results were obtained with CD117 and toluidine blue [5]. However, there exists no standardization for counting them.

MC counting

MC quantification was performed using a hotspot method, similar to approaches used in tumor angiogenesis studies, using a Labomed LX 300 microscope (Labomed, Inc., Los Angeles, CA). Each toluidine blue-stained slide was initially scanned at low magnification (10× eyepiece and 10× objective, total magnification 100×) to identify areas with the highest MC density. From each slide, three non-overlapping hotspot areas were selected. MCs were then counted at high-power magnification with a 40× objective and a 10× eyepiece (total magnification 400×) in 10 consecutive high-power fields (HPFs) within each hotspot, and the mean value was recorded. The high-power field diameter was 0.45 mm, corresponding to a field area of 0.159 mm². All slides were evaluated under identical optical settings to maintain consistency.

Degenerative changes were assessed on hematoxylin- and eosin-stained sections, and MCs were counted on toluidine blue sections. Hyaline degeneration was defined as the presence of homogeneous, eosinophilic, acellular material replacing smooth muscle bundles without evidence of necrosis or a significant inflammatory infiltrate. No other types of degeneration (cystic, red, or calcific) were identified in the present study. The number of MCs/10 HPF in leiomyomas with degeneration was recorded.

All slides were evaluated by a single pathologist to avoid interobserver variability.

Statistical analysis

Data were entered into Microsoft Excel 2013 (Microsoft Corporation, Redmond, WA, USA) and analyzed using IBM Corp. Released 2011. IBM SPSS Statistics for Windows, Version 17. Armonk, NY: IBM Corp. Continuous variables were expressed as mean ± standard deviation (SD), and categorical variables were presented as frequencies and percentages. Data distribution was assessed for normality prior to the application of parametric tests.

The mean MC counts per 10 HPF were compared across age groups and sites of leiomyoma using one-way analysis of variance (ANOVA). Post-hoc analysis was performed where applicable.

Comparison of MC counts between leiomyoma and adjacent myometrium was performed using a paired Student’s t-test, as the samples were derived from the same specimen. The association between MC density categories and age groups was analyzed using the chi-square test. Karl Pearson’s correlation coefficient was used to assess the relationship between MC counts in leiomyoma and adjacent myometrium. A p-value of < 0.05 was considered statistically significant at a 95% confidence level. Fisher’s exact test was used to study the association between leiomyoma with degeneration and MC counts.

## Results

**Figure 1:**
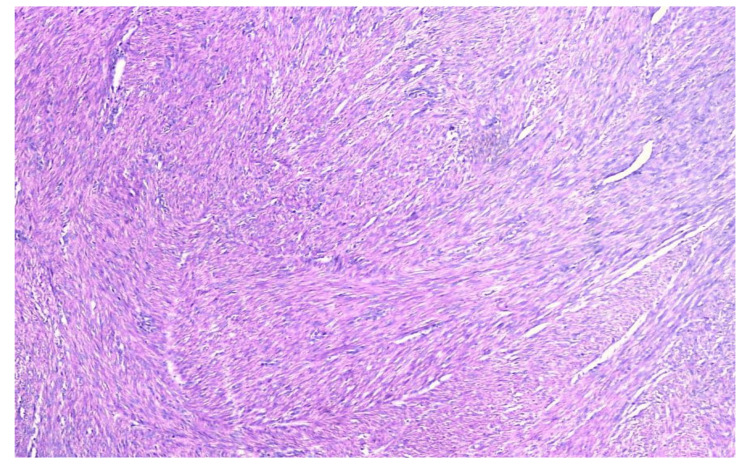
Leiomyoma without degeneration (H&E, 100× magnification)

A total of 100 hysterectomy specimens with uterine leiomyoma were analyzed. The cases were evaluated with respect to age distribution, site of origin, MC counts in leiomyomas, and comparison with adjacent myometrium. Hematoxylin- and eosin-stained slides of leiomyomas without degeneration showed well-circumscribed neoplasms arranged in interlacing fascicles of spindle cells with a moderate amount of eosinophilic cytoplasm and cigar-shaped nuclei (Figure 1).

**Figure 2:**
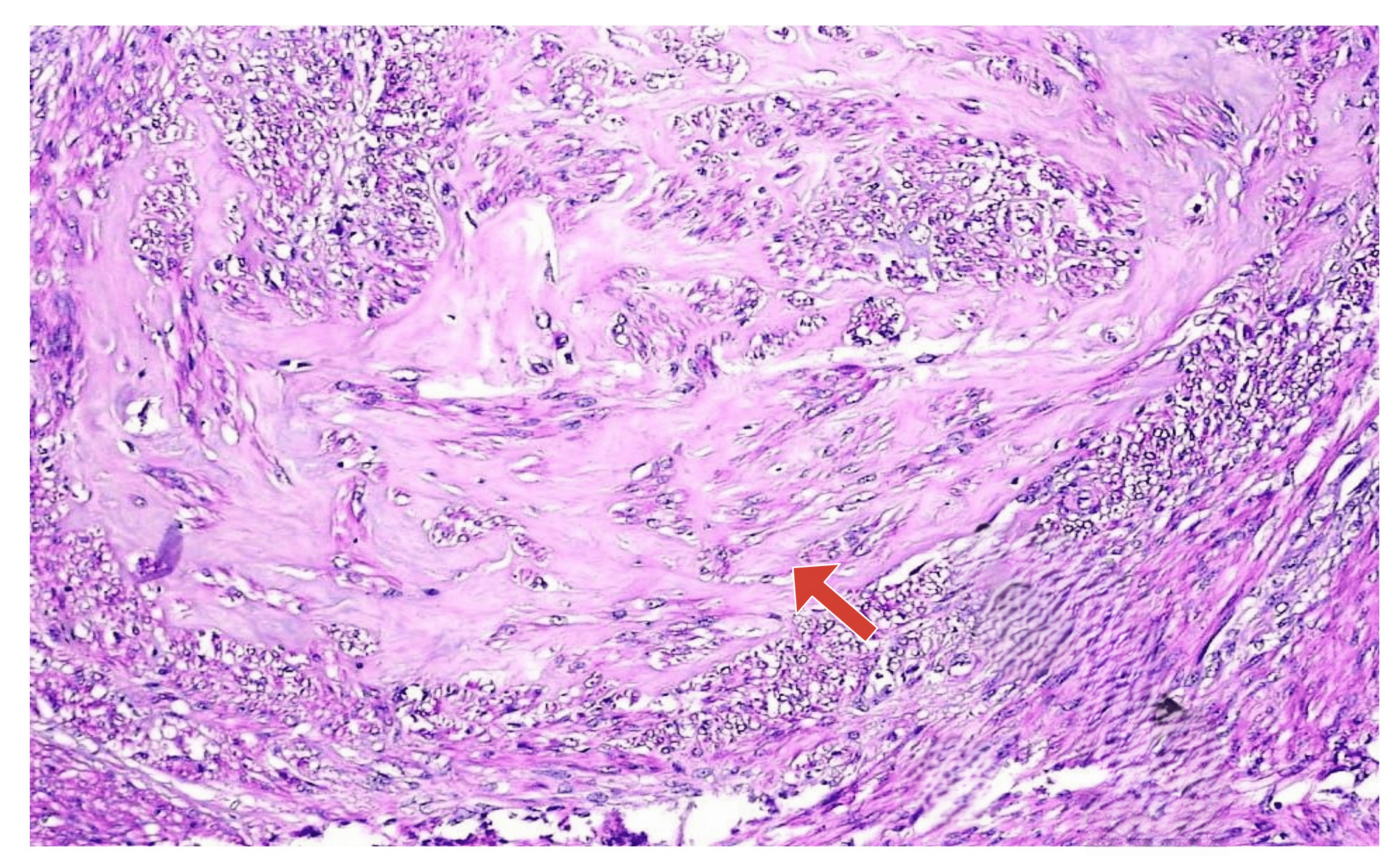
Leiomyoma with degeneration—the arrow indicates hyaline degeneration (H&E, 100× magnification)

Sections of leiomyomas with hyaline degeneration showed interlacing fascicles of spindle cells separated by homogenous, eosinophilic collagenous material. No necrosis, cellular atypia, or mitosis was noted (Figure 2).

**Figure 3:**
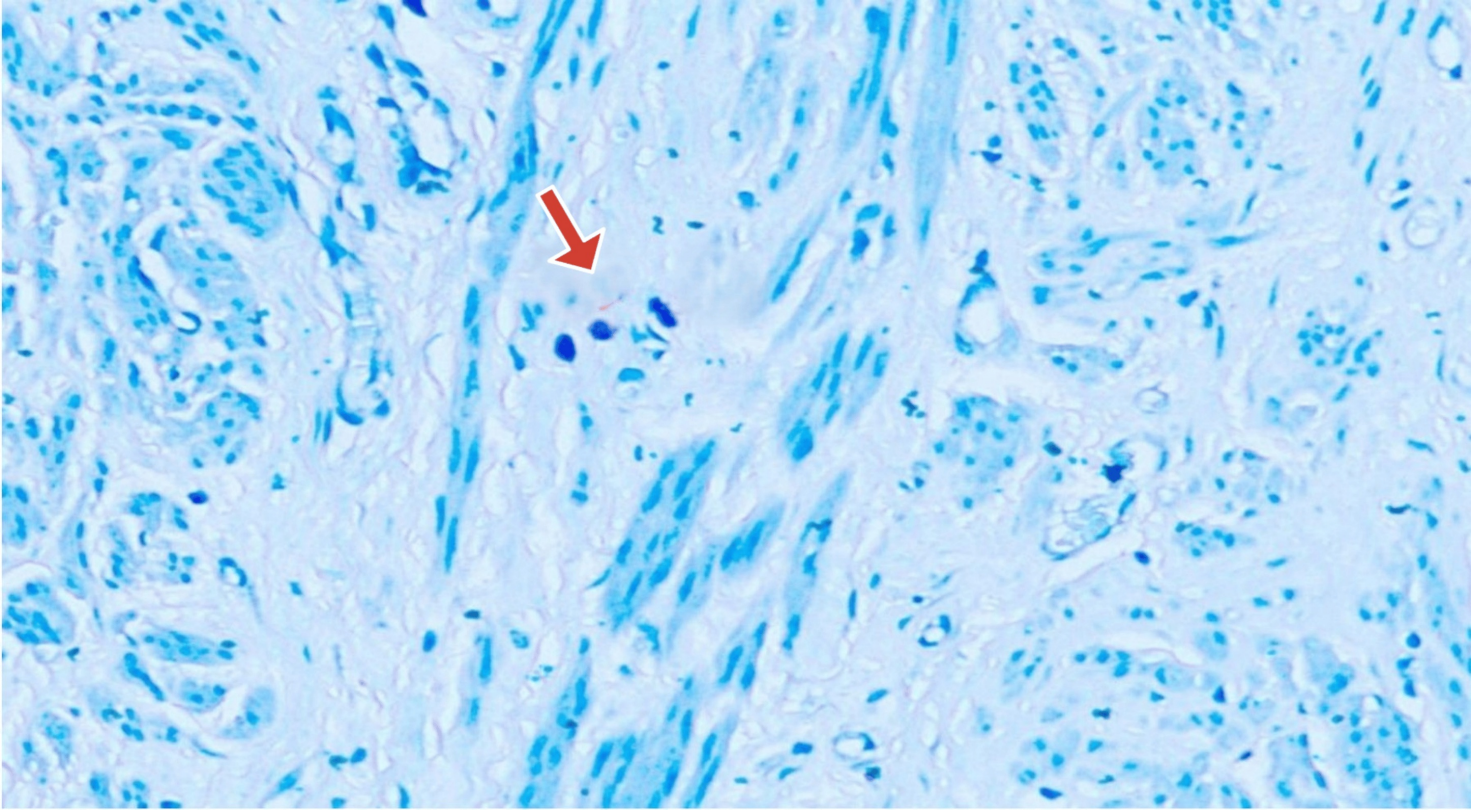
Myometrium with mast cells (1% toluidine blue, 400× magnification)

Microscopic examination of toluidine blue-stained slides revealed MCs, which were round-to-oval cells with inconspicuous nuclei and coarse, violet metachromatic cytoplasmic granules (Figure 3).

**Figure 4:**
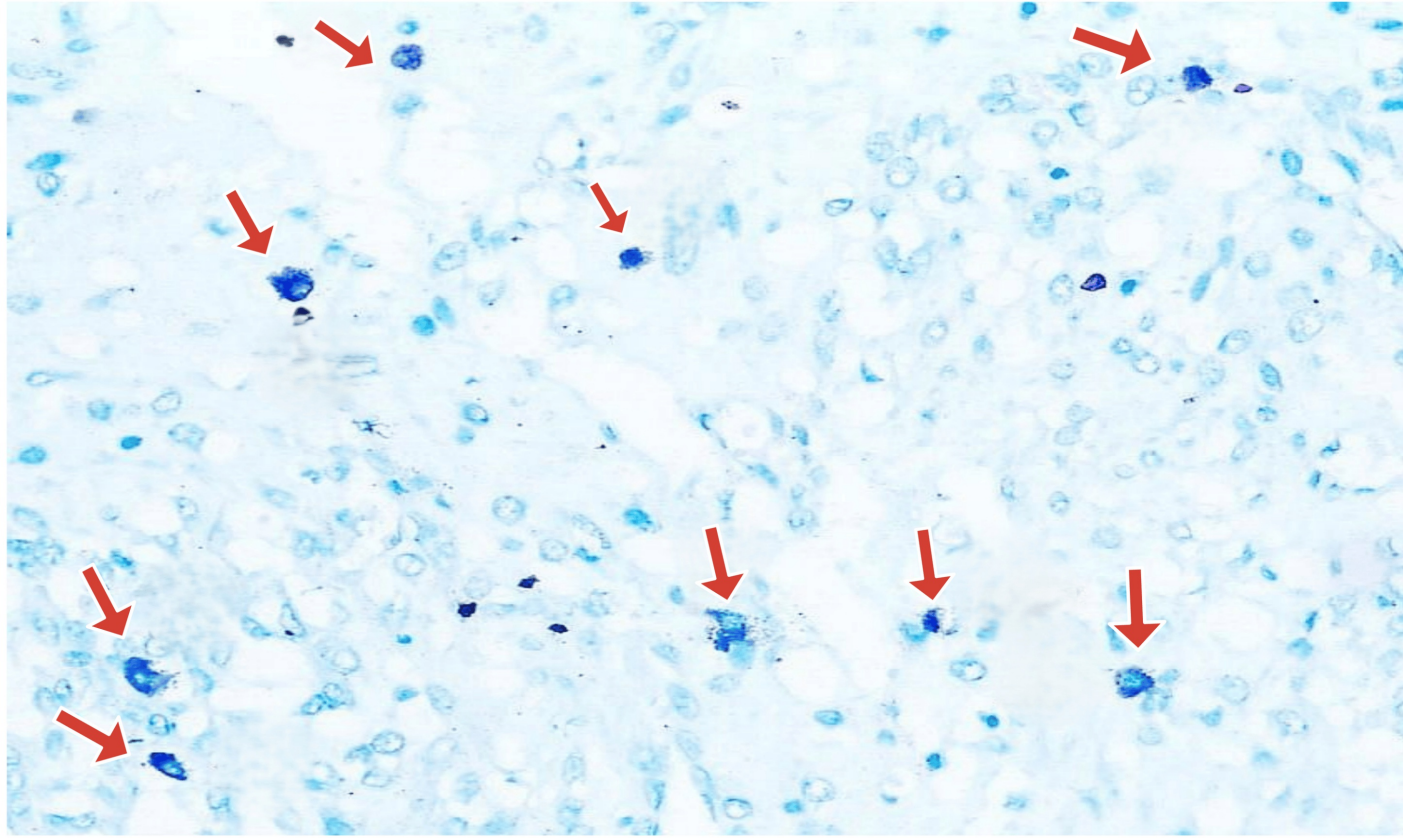
Leiomyoma without degeneration—high-power view of a hotspot area with nine mast cells in one field (1% toluidine blue, 400× magnification)

**Figure 5:**
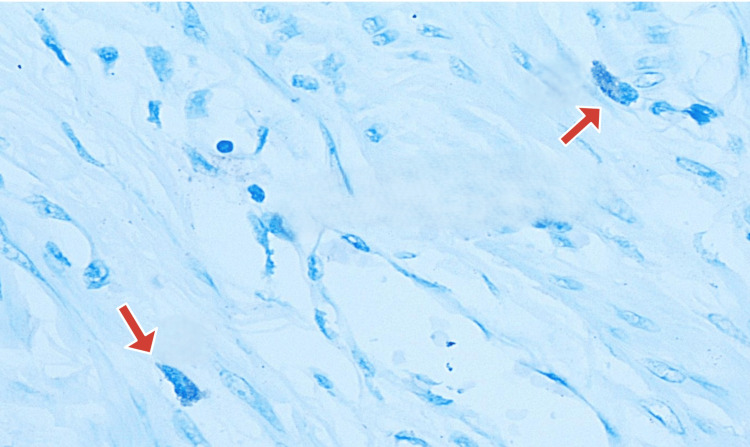
Leiomyoma with hyaline degeneration—high-power view of a hotspot area with two mast cells in one field (1% toluidine blue, 400× magnification)

Hotspot areas, i.e., areas where maximum MCs were seen, were identified under low-power magnification and then were counted under high power (400× magnification) for 10 consecutive fields in each slide. This was labeled as "MC counts/10 HPF" (Figures 4, 5).

Age distribution

Uterine leiomyomas showed a perimenopausal predominance, with 49% of cases occurring in the 40-50-year age group. The mean age of patients was 45.94 ± 6 years; 18% of cases were in the reproductive age group (<40 years), while 33% were postmenopausal (>50 years) (Table 1).

**Table 1:** Distribution of leiomyomas according to age

Age Group (Years)	Number of Cases (n)	Percentage (%)
<40	18	18
40–50	49	49
>50	33	33
Total	100	100

Site distribution

The bulk of leiomyomas in this study, i.e., 59%, were found to be intramural in location, while submucosal leiomyomas constituted the smallest volume of cases (15%) (Table 2).

**Table 2:** Distribution of leiomyomas according to site

Site	Number of Cases (n)	Percentage (%)
Intramural	59	59
Subserosal	26	26
Submucosal	15	15
Total	100	100

MC counts in the adjacent myometrium

The mean MC count per 10 HPF in adjacent myometrium was highest in the reproductive age group (17.59 ± 9.02), followed by the perimenopausal group (14.94 ± 6.39) and lowest in the postmenopausal group (14.06 ± 5.66). This difference across age groups was modest but statistically significant (ANOVA, p = 0.0458, F-value = 3.193), with higher MC counts noted in the younger age group (Table 3).

**Table 3:** Distribution of mast cells (MC)/10 HPF in the myometrium across age groups ANOVA: analysis of variance test applied; MC: mast cells; HPF: high-power field

Age Group	n	Mean MC/10 HPF	SD	Min MC/10 HPF	Max MC/10 HPF	p-value	F-value
<40	18	17.59	9.02	8	36	0.0454	3.193
40–50	49	14.94	6.39	5	36		
>50	33	14.06	5.66	6	28		

MC counts in leiomyoma across age groups

The mean MC count per 10 HPF in leiomyomas was 40.76 ± 23.14 in the reproductive age group, 30.88 ± 12.26 in the perimenopausal group, and 28.62 ± 9.68 in the postmenopausal group. The difference was statistically highly significant (ANOVA, p = 0.0005, F-value = 8.166), indicating a significant inverse relationship between MC count and advancing age (Table 4).

**Table 4:** Distribution of mast cells (MC)/10 HPF in leiomyoma across age groups MC: mast cells; ANOVA: analysis of variance (ANOVA) test applied

Age Group	n	Mean MC/10 HPF	SD	Min	Max	p-value	F-value
<40	18	40.76	23.14	20	88	0.0005	8.166
40–50	49	30.88	12.26	18	86		
>50	33	28.62	9.68	17	48		

MC counts were categorized as <20, 20-40, and >40 for chi-square analysis. Chi-square analysis also demonstrated a significant association between MC count categories and age groups (chi-square = 9.789, p = 0.0442) (Table 5).

**Table 5:** Association between mast cell count categories and age groups Chi-square test applied MC: mast cells; HPF: high-power field

Age Group (Years)	<20 MC/10 HPF	20–40 MC/10 HPF	>40 MC/10 HPF	Total (n)	Chi-square (χ²)	Degrees of Freedom (df)	p-value
<40	0	6	12	18	9.787	4	0.0442
40–50	6	28	15	49			
>50	7	20	6	33			
Total	13	54	33	100			

MC counts according to the site of leiomyoma

The mean MC count per 10 HPF was highest in intramural leiomyomas (45.76), followed by subserosal (34.88) and submucosal (28.62) leiomyomas. This difference was statistically significant (ANOVA, p = 0.0054, F-value = 5.505) (Table 6).

**Table 6:** Distribution of mast cells in leiomyomas according to the site of origin MC/10 HPF: mast cells per 10 high-power fields; ANOVA: analysis of variance test applied

Site	n	Mean MC/10 HPF	SD	Min	Max	p-value	F-value
Intramural	59	45.76	21.48	24	88	0.0054	5.505
Subserosal	26	34.88	20.87	22	60		
Submucosal	15	28.62	9.89	20	40		

Comparison between leiomyoma and adjacent myometrium

The distribution pattern showed a rightward shift in MC counts in leiomyoma compared with the adjacent myometrium. While the majority of myometrial samples (65%) exhibited fewer than 20 MC per 10 HPF, nearly one-third of leiomyomas demonstrated counts exceeding 40 per 10 HPF.

A paired Student's t-test demonstrated that MC counts were significantly higher in leiomyomas compared to adjacent myometrium (t = 18.65, p < 0.001). A strong positive correlation was observed between MC counts in leiomyoma and adjacent myometrium (Pearson’s correlation coefficient, r = 0.886, p < 0.001) (Table 7).

**Table 7:** Distribution of mast cells in leiomyoma and adjacent myometrium

Mast Cells/10 HPF	Leiomyoma (n=100)	%	Myometrium (n=100)	%	p-value	t-value
<20	33	33	65	65	<0.001	18.654
20–40	38	38	35	35		
40–60	24	24	0	0		
60–80	2	2	0	0		
80–100	3	3	0	0		
Total	100	100	100	100		

MC counts in leiomyoma with and without degeneration

Of the 100 cases, 10 showed hyaline degeneration; 80% of leiomyomas with degeneration had fewer than 20 MCs/10 HPF, while none of the leiomyomas without degeneration fell into this lowest category.

Fisher's exact test performed on this data showed a statistically significant association between degeneration and lower MC counts (p < 0.001) (Table 8).

**Table 8:** Distribution of mast cells in leiomyomas with degeneration and without degeneration Fisher's exact test applied MC: mast cells; HPF: high-power field

MC/10 HPF	Cases With Degeneration (n=10)	%	Cases Without Degeneration (n=90)	%	Fisher’s Exact p-value
<20	8	80	0	0	<0.001
20–40	2	20	61	67.78	
>40	0	0	29	32.22	

## Discussion

There is scarce literature regarding the distribution and potential relationship between MC and various conditions of the myometrium, with several authors disagreeing with the conclusions. The human uterus is considered relatively rich in MCs compared to other tissues of the body. The present study was undertaken to evaluate the distribution of MCs in uterine leiomyomas, to compare their density with that of the adjacent myometrium, and to analyze their association with clinicopathological parameters.

MCs were stained with toluidine blue, a metachromatic stain, which provided good contrast and stained the granules bright purple-pink. However, there exists no standardization regarding the selection of hotspot areas for MC counting. Therefore, the slides were all evaluated by a single pathologist to avoid interobserver variability.

In our study, uterine leiomyomas showed a perimenopausal age predominance, with 49% of the cases in the ages between 40 and 50 years, the mean age being 45.94 years. These findings were concurrent with those of Ulin et al. [6], Delamater and Santoro [7], and Wise and Laughlin-Tommaso [8], who reported that uterine fibroids peak in the perimenopausal years and decline following menopause. Studies done by Casini et al. [9], Pritts et al. [10], and Gousuddin et al. [11] reported intramural to be the most common site of origin of uterine leiomyomas, which was consistent with the findings of this study, in which 59% of the cases were intramural in origin.

There exists no consensus regarding the minimum number of MCs that should be present in uterine leiomyomas or the adjacent myometrium, with different authors proposing varied ranges. In the myometrium, mean MC counts/10 HPF were found to be the highest in the reproductive age group (17.59/10 HPF) and the lowest in the postmenopausal age group (14.06/10 HPF). A similar finding was emphasized by Mori et al. [3] and Drudy et al. [12], who found that the lowest number of MCs was seen after menopause.

An inverse correlation was found between the MC count per 10 HPF in leiomyomas and increasing age in the present study. The greatest MC count of 88 cells/10 HPF in leiomyoma was found in the reproductive age group. These findings were similar to those of Maluf and Gersell [13] and Orii et al. [14], who observed a decreasing trend in the mean number of MCs with advancing age in leiomyomas.

The distribution of MC in leiomyoma according to site of origin was studied, and the maximum number was found in the intramural leiomyoma. This was synchronous with the findings of Gousuddin et al. [11], who concluded that intramural leiomyoma showed the highest MC count.

Our study revealed an approximate twofold increase in the distribution of MC in uterine leiomyoma in comparison with the adjacent myometrium, which was statistically significant. In 65% of the cases, the myometrium had fewer than 20 MC/10 HPF. These observations were consistent with those of multiple authors, including Maluf and Gersell [13], who reported that MC counts were consistently higher in leiomyomas than in the myometrium, with ratios ranging from 3:1 to 12:1.

Despite the presence of various degenerations in uterine leiomyomas, only hyaline degeneration was observed in our study. Of the 100 cases of leiomyomas, only 10 had degeneration, and 80% of leiomyomas with degeneration showed a much lower MC count (<20 cells/10 HPF) as compared to a majority (67.78%) of leiomyomas without degeneration, which revealed average MC counts in the range of 20-40 cells/10 HPF. These findings were in conformity with those of Abeyratne NVA et al., who reported that MC counts were low in leiomyomas with hyaline degeneration [5].

This study has several important strengths. First, the paired tissue comparison design, in which leiomyoma tissue was compared with adjacent myometrium from the same specimen, is methodologically robust. This approach minimizes inter-patient biological variability, including hormonal influences and systemic confounders, thereby strengthening internal validity. Second, the sample size of 100 cases (despite the constraints posed by COVID-19) is substantial for a single-center histopathological study and enhances the statistical reliability of subgroup analyses. Third, multiple complementary statistical methods were employed, including paired t-tests, ANOVA, Fisher’s exact test, and Pearson correlation analysis. This comprehensive analytical approach allowed for rigorous evaluation of both continuous and categorical associations, thereby improving the robustness of the findings.

Nevertheless, certain limitations must be acknowledged. As a retrospective observational study, the design is inherently subject to selection bias and limited control over potential confounding variables. MC quantification was performed using hotspot-based assessment, which, although commonly utilized in histopathological studies, may introduce focal sampling bias and affect reproducibility. Furthermore, slide evaluation was conducted by a single observer without formal assessment of intraobserver or interobserver reliability, which may introduce observational bias. Blinding to clinical and histopathological variables was not performed, potentially influencing the assessment. The number of leiomyomas with degeneration was relatively small (n = 10), limiting the generalizability of conclusions regarding degenerative changes. As this was a cross-sectional observational study, the findings demonstrate associations rather than causation. Finally, as this was a single-center study conducted on a defined regional population, the generalizability of findings to other demographic groups may be limited.

## Conclusions

The present study showed a significant increase in MC counts in uterine leiomyoma compared with the adjacent myometrium, with higher counts observed in reproductive-age patients and in leiomyomata without degeneration. MC counts were observed to be higher in intramural leiomyomata. A significant inverse association of leiomyoma with advancing age was also demonstrated. The reduced MC counts observed in leiomyoma with degeneration suggest that MC density varies with tumor characteristics.

The findings in this study highlight a probable relationship between MC counts and the stromal microenvironment of uterine leiomyomata. However, being an observational study, the results reflect associations rather than a direct causal relationship. Further mechanistic studies may be done to elucidate the functional significance of MC in leiomyoma pathogenesis.
